# IncHI1 plasmids are epidemic vectors that mediate transmission of *tet*(X4) in *Escherichia coli* isolated from China

**DOI:** 10.3389/fmicb.2023.1153139

**Published:** 2023-05-25

**Authors:** Yan Zhang, Jie Zhang, Ping Cai, Yang Lu, Ruan-Yang Sun, Meng-Tao Cao, Xiao-Li Xu, Mark A. Webber, Hong-Xia Jiang

**Affiliations:** ^1^Guangdong Provincial Key Laboratory of Veterinary Pharmaceutics Development and Safety Evaluation, College of Veterinary Medicine, South China Agricultural University, Guangzhou, China; ^2^Guangdong Laboratory for Lingnan Modern Agriculture, Guangzhou, China; ^3^Instrumental Analysis and Research Center, South China Agricultural University, Guangzhou, China; ^4^Quadram Institute Bioscience, Norfolk, United Kingdom

**Keywords:** tigecycline, AMR, conjugation, plasmid, ISCR2

## Abstract

**Introduction:**

This study aimed to investigate the genetic factors promoting widespread Q6 dissemination of *tet*(X4) between Escherichia coli and to characterize the genetic contexts of *tet*(X4).

**Methods:**

We isolated E. coli from feces, water, soil and flies collected across a large-scale chicken farm in China in 2020. Antimicrobial susceptibility testing and PFGE typing were used to identify tigecycline resistance and assess clonal relationships among isolates. Plasmids present and genome sequences were analyzed by conjugation, S1 pulsed-field gel electrophoresis (PFGE), plasmid stability testing and whole-genome sequencing.

**Results:**

A total of 204 tigecycline-resistant E. coli were isolated from 662 samples. Of these, we identified 165 *tet*(X4)-carrying E. coli and these strains exhibited a high degree of multidrug resistance. Based on the geographical location distribution of the sampled areas, number of samples in each area and isolation rate of tigecycline-resistant strains and *tet*(X4)-carrying isolates, 72 *tet*(X4)-positive isolates were selected for further investigation. Tigecycline resistance was shown to be mobile in 72 isolates and three types of *tet*(X4)-carrying plasmids were identified, they were IncHI1 (n = 67), IncX1 (n = 3) and pO111-like/IncFIA(HI1) (n = 2). The pO111-like/IncFIA(HI1) is a novel plasmid capable of transferring *tet*(X4). The transfer efficiency of IncHI1 plasmids was extremely high in most cases and IncHI1 plasmids were stable when transferred into common recipient strains. The genetic structures flanked by IS1, IS26 and ISCR2 containing *tet*(X4) were complex and varied in different plasmids.

**Discussion:**

The widespread dissemination of tigecycline-resistant *E. coli* is a major threat to public health. This data suggests careful use of tetracycline on farms is important to limit spread of resistance to tigecycline. Multiple mobile elements carrying *tet*(X4) are in circulation with IncHI1 plasmids the dominant vector in this setting.

## Introduction

The global transmission of multidrug resistant bacteria is a major threat to public health (Runcharoen et al., 2017). Tigecycline is an important antibiotic which can be used as a “last line of defense” against serious infections caused by multidrug-resistant (MDR) Gram-negative bacteria (Doan et al., 2006) which was launched for clinical usage in 2010 in China. Since then, it has been used increasingly in treating multidrug-resistant bacterial infections (Zhang et al., 2020a). However, owing to the increased use of tigecycline, including for treatment of carbapenem-resistant Enterobacteriaceae infections, tigecycline-resistant bacteria have emerged and now pose an important clinical concern (Kaewpoowat and Ostrosky-Zeichner, 2014; Ni et al., 2016).

Tigecycline resistance mainly results from three mechanisms: tetracycline-specific efflux proteins, ribosomal protection proteins, or enzymatic inactivation of tetracycline (Sun et al., 2013; Linkevicius et al., 2016). Among these, enzymatic inactivation is the most common resistance mechanism in bacteria. A tetracycline modifying enzyme-*tet*(X) was first reported in anaerobes, but plays an important role in many bacteria, including *E. coli* (Speer et al., 1991; Chopra and Roberts, 2001; Moore et al., 2005).

The wide transmission of plasmid-mediated *tet*(X) in recent years presents a threat for clinical treatment of infections (Zhai et al., 2022), mutation of *tet*(X) into different variants has been observed with extended spectrum of activity of some conferring resistance to tigecycline (Yaghoubi et al., 2022). Among the different subtypes of *tet*(X), *tet*(X3)/*tet*(X4)/*tet*(X5) can mediate high-level resistance to tigecycline which is a clinical problem (Zhang et al., 2020a). Of these, *tet*(X4) has recently been detected from food-producing animals and is the most prevalent *tet*(X) subtype in China (Sun C. et al., 2019; Feng et al., 2022; Wu et al., 2022). The emergence of tigecycline resistance in animals suggests this may be an important reservoir for human infection, recent reports indicate that the transmission of *tet*(X4) can be mediated by diverse vectors, including IncX1, IncX4, IncX1/N, IncX1/FIA/B/Y, IncX1/FIA/B/HI1A/B, IncFIA/B/HI1A/B, IncFII, Col, IncQ, and IncFIB/FIA(HI1)/X1 plasmids (Sun C. et al., 2019; Sun et al., 2020; Zhang et al., 2020b; Mohsin et al., 2021; Shafiq et al., 2022). This suggests *tet*(X4) genes can be spread by many vectors, but it is unclear which is of most importance. In this study, we investigated mechanisms of transmission of *tet*(X4) obtained from a single large chicken farm. Combining this data with the literature, we provide firm evidence for the growing problem of tigecycline resistance in the farm environment and characterize the dominant vectors responsible for transmission.

## Materials and methods

### Bacterial strains and antimicrobial susceptibility testing

A total of 662 samples were recovered from 10 areas across a large-scale chicken farm in Guangdong Province, China in August 2020, 526 were collected from feces, 47 were collected from water, 81 were collected from soil and 8 samples were collected from flies (Supplementary Figure S1). Tigecycline-resistant *E. coli* were isolated using MacConkey agar containing 4 mg/L tigecycline. The presence of *tet*(X4) was identified by PCR using primers *tet*(X4)-F (ATCGTTATGGGTAATGGAC) and *tet*(X4)-R (ACCTGGTAAGAAGAAGTGG) (495 bp). In addition, a *tet*(X2/3/5) universal PCR test (forward: 5’-CTGTCCTGGATTTTTTCAG-3′; reverse 5’-TTCTTTGTAGCGTTCGTC-3′) was used to identify the presence of other *tet*(X) variants. The following strain nomenclature was used in this study: area of the farm/serial number of samplings (e.g., 19A/20). Seventy-two *tet*(X4)-carrying strains were selected for subsequent experiments from all *tet*(X4)-carrying *E. coli* strains in this study to explore the transmission mechanisms of *tet*(X4). These 72 strains were chosen based on the geographical location distribution of the sampled areas, number of samples in each area and isolation rate of tigecycline-resistant strains and *tet*(X4)-carrying isolates to ensure the reliability of the results.

Antimicrobial susceptibility testing was performed by agar dilution method according to the CLSI guidelines (CLSI, 2019). The following 12 antibiotics were tested: tigecycline (TGC), ciprofloxacin (CIP), tetracycline (TET), meropenem (MEM), cefotaxime (CTX), ampicillin (AMP), fosfomycin (FOS). doxycycline (DOX), minocycline (MIN), amikacin (AMI), enrofloxacin (ENO), florfenicol (FLF). Susceptibility profiles to colistin (CL) were determined by the broth microdilution method. *E. coli* ATCC25922 was used as a quality control strain.

### Pulsed-field gel electrophoresis

The 72 tested isolates were subjected to PFGE analysis using *Xba*I restriction enzyme to analyze the diversity of *tet*(X4)-carrying *E. coli* isolates to assess the relationship between different isolates. Bacterial DNA samples were electrophoresed with pulse times from 2.2 s to 54.2 s over 19 h and PFGE was performed at 14°C in 0.5 × TBE. Isolates with a profile possessed >80% similarity were considered to belong to the same PFGE group (Stanley et al., 1995). *Salmonella enterica* H9812 was used as PFGE marker. PFGE patterns were compared using the BioNumerics software (Applied Maths, Sint-Martens-Latem, Belgium).

### Characterization and stability of *tet*(X4)- harboring plasmids

To investigate the transfer ability of plasmids isolated from 72 tested *E. coli* strains, filter mating was performed with *E. coli* C600 as a recipient. The recipient and donor strains were combined 1:3/1:1/3:1 on a filter disk placed on LB agar the mating mixture was incubated at 37°C overnight. Transconjugants were selected on MacConkey agar containing tigecycline (4 mg/L) and streptomycin (2,000 mg/L) following incubation (Liu et al., 2018). Enterobacterial Repetitive Intergenic Consensus (ERIC) PCR typing and the *tet*(X4) target PCR of the transconjugants/donors/recipients were subsequently performed to confirm whether the plasmid was successfully transferred to the recipient (Sun J. et al., 2019). Non-conjugative plasmids were extracted and transformed into competent DH5α by electroporation. The transfer efficiency represents the fraction of potential recipient cells which receive a plasmid. Plasmid PCR-based replicon typing (PBRT) and PFGE using S1 nuclease (S1-PFGE) were performed as previously described (Barton et al., 1995; Carattoli et al., 2005) to determine the plasmid incompatibility type and size of *tet*(X4)-carrying plasmids. Two additional replicon primers (pO111-F: 5’-TTCCTGCCGTTTTTTATCT-3′, pO111-R: 5’-CTGCCATTCTCCGAGTTTG-3′; IncX1-F: 5’-GCTTAGACTTTGTTTTATCGTT-3′, IncX1-R: 5’-TAATGATCCTCAGCATGTGAT-3′) were included.

The stability of plasmids was studied as previously described (Sandegren et al., 2012). We selected 3 *E. coli* strains as recipients to generate transconjugants containing *tet*(X4)-carrying IncHI1 plasmids. These strains were reference strains C600 and MG1655 and a duck isolate BYZ3 (which contains no plasmids). IncHI1 plasmid-carrying transconjugants were grown overnight at 37°C in LB broth without any antibiotics. The following day the strains were passaged by 1,000-fold dilution into fresh medium and were allowed to grow for another 24 h. The same procedure was repeated, and the strains were cultured for 7 days. After day 7, cultures were serially diluted (10-fold) in saline (NaCl [0.9%, wt/vol]) and plated on LB agar plates. Single colonies (n = 100) were picked, and the loss of plasmids was confirmed by the *tet*(X4) and IncHI1 replicon target PCR. Three parallel samples were analyzed for each strain.

### WGS and bioinformatics analyses

Based on results of PFGE, type of *tet*(X4)-carrying plasmid and isolation source of sample, six strains to represent the diversity in the panel were selected for whole genome sequencing. Three of them carrying a transferable IncHI1-*tet*(X4) plasmid (2 strains isolated from feces samples, 1 strain isolated from soil sample), the other three strains carrying untransferable *tet*(X4)-carrying plasmids (1 strain isolated from feces sample, 2 strains isolated from soil samples).

Genomic DNA of the 6 *E. coli* strains was extracted by Hipure Bacterial DNA kit (Qiagen, Hilden, Germany). The genomes were sequenced using both long-read Nanopore and short-read Illumina Miseq sequencing platforms. The clean reads were then used to create hybrid assemblies using the CLC Genomics Workbench. Genes and insertion element confirmation and annotation were done with RAST,^1^ resfinder,^2^ and ISfinder.^3^ Plasmid replicon type was analyzed using the plasmidfinder tool.^4^ Comparison figures of genetic structures and multiple plasmids were generated by Easyfig (Sullivan et al., 2011), BRIG (Alikhan et al., 2011) and adapted using Adobe Illustrator Adobe Illustrator.

## Results

### Identification, antibiotic susceptibility and PFGE analysis of *tet*(X4)-carrying *Escherichia coli*

A total of 204 tigecycline-resistant *E. coli* strains were isolated and identified from a large chicken farm. The tigecycline resistant isolates contained 165 *tet*(X4)-carrying *E. coli*, including 110 strains isolated from feces samples, and 55 strains isolated from soil samples (Supplementary Table S1). Of 39 tigecycline-resistant *E. coli* strains not harboring *tet*(X4), 22 isolates carried *tet*(X2/3/5) according to PCR with these universal primers. Of the 165 *tet*(X4)-carrying *E. coli* isolates, 72 were selected to represent each region of the chicken farm (feces: *n* = 53; soil: *n* = 19) for subsequent experiments (Supplementary Table S2).

**Figure 1:**
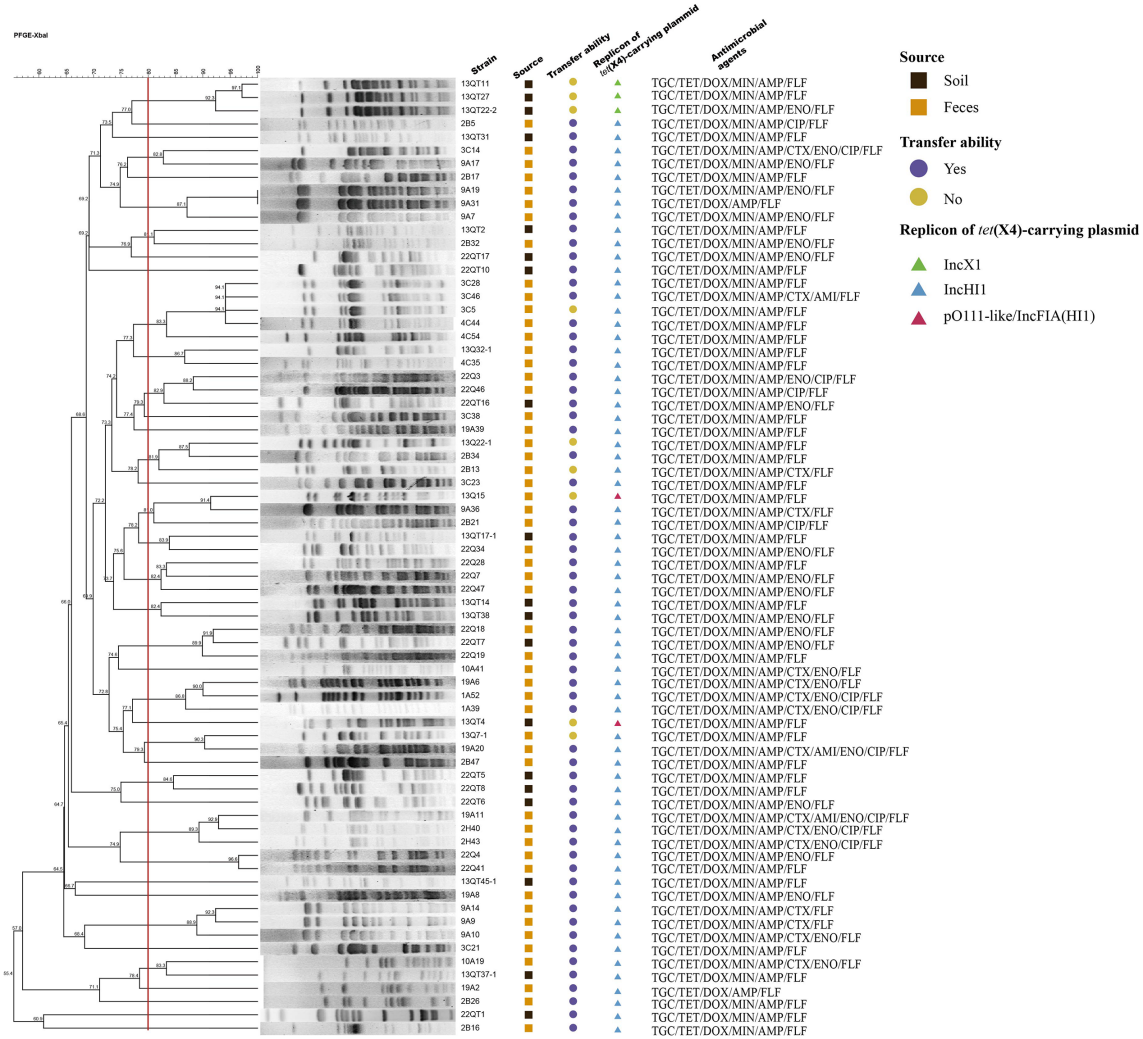
The PFGE profiles, plasmid characteristics and antibiotic-resistant profiles of 72 strains. The boxes represent the source of strains (Dark boxes: strains were isolated from the soil samples; Dark yellow boxes: strains were isolated from the feces samples). The circles represent the transfer ability of *tet*(X4)-carrying plasmids (Purple circles: plasmids can be transferred by conjugation; Brown circles: plasmids cannot be transferred by conjugation). The triangles represent the replicon of *tet*(X4)-carrying plasmids (Green triangles: IncX1 plasmid; Blue triangles: IncHI1 plasmid; Red triangles: pO111-like/IncFIA(HI1) plasmid).

Tigecycline MICs for test strains ranged from 8 to 16 mg/L and they were completely resistant to tetracycline, doxycycline, ampicillin and florfenicol. Seventy one strains (98.61%) were resistant to minocycline. The resistance rates to enrofloxacin, ciprofloxacin, cefotaxime and amikacin were 38.89% (28/72), 13.89% (10/72), 22.22% (16/72), and 4.17% (3/72), respectively (Figure 1). All strains were fully susceptible to meropenem, colistin and fosfomycin.

The *E. coli* isolates were divided into 39 different PFGE clusters with 80% genetic similarity. Based on the PFGE results, it was concluded that horizontal transmission of *tet*(X4) in the large chicken farm of this study is common (Figure 1).

### Analysis of *tet*(X4)-carrying plasmids

A total of 63 transconjugants and 9 transformants were obtained from the 72 isolates and all of them contained independent *tet*(X4)-carrying plasmids that ranged in size from 45 to 250 kb. IncHI1 plasmids were by far the most prevalent type (*n* = 67) as assessed by PCR replicon typing demonstrating these plasmids are the dominant vectors of *tet*(X4) in this setting. The remaining five plasmids were IncX1 (*n* = 3) and pO111-like/IncFIA(HI1) (pO111-like with additional IncFIA/HI1 replicons) (*n* = 2) (Figure 1).

The transfer efficiency of 27 IncHI1 plasmids was determined and was generally high, ranging between 1.0 × 10^−7^ and 3.4 × 10^−1^. The transfer efficiency of four IncHI1 plasmids was found to be extremely high (above 1.0 × 10^−1^) (Supplementary Table S4). The stability of six IncHI1 plasmids was tested in different hosts. These were stably maintained for 7 days in C600, interestingly, a gradual loss of the tested plasmids was observed with none of the population maintaining the plasmids after 7 days of passage in MG1655 and BYZ3 (Supplementary Figure S2).

### Sequence analysis of *tet*(X4)-carrying plasmids

Six *E. coli* strains carrying *tet*(X4) plasmids (19A20, 13QT31, 22Q34, 13QT4, 13Q15, and 13QT11) were fully sequenced and annotated. The *tet*(X4)-carrying plasmid types were IncHI1 (*n* = 3), pO111-like/IncFIA(HI1) (*n* = 2) and IncX1 (*n* = 1), respectively. See Supplementary Table S3 for sequence accession numbers.

**Figure 2:**
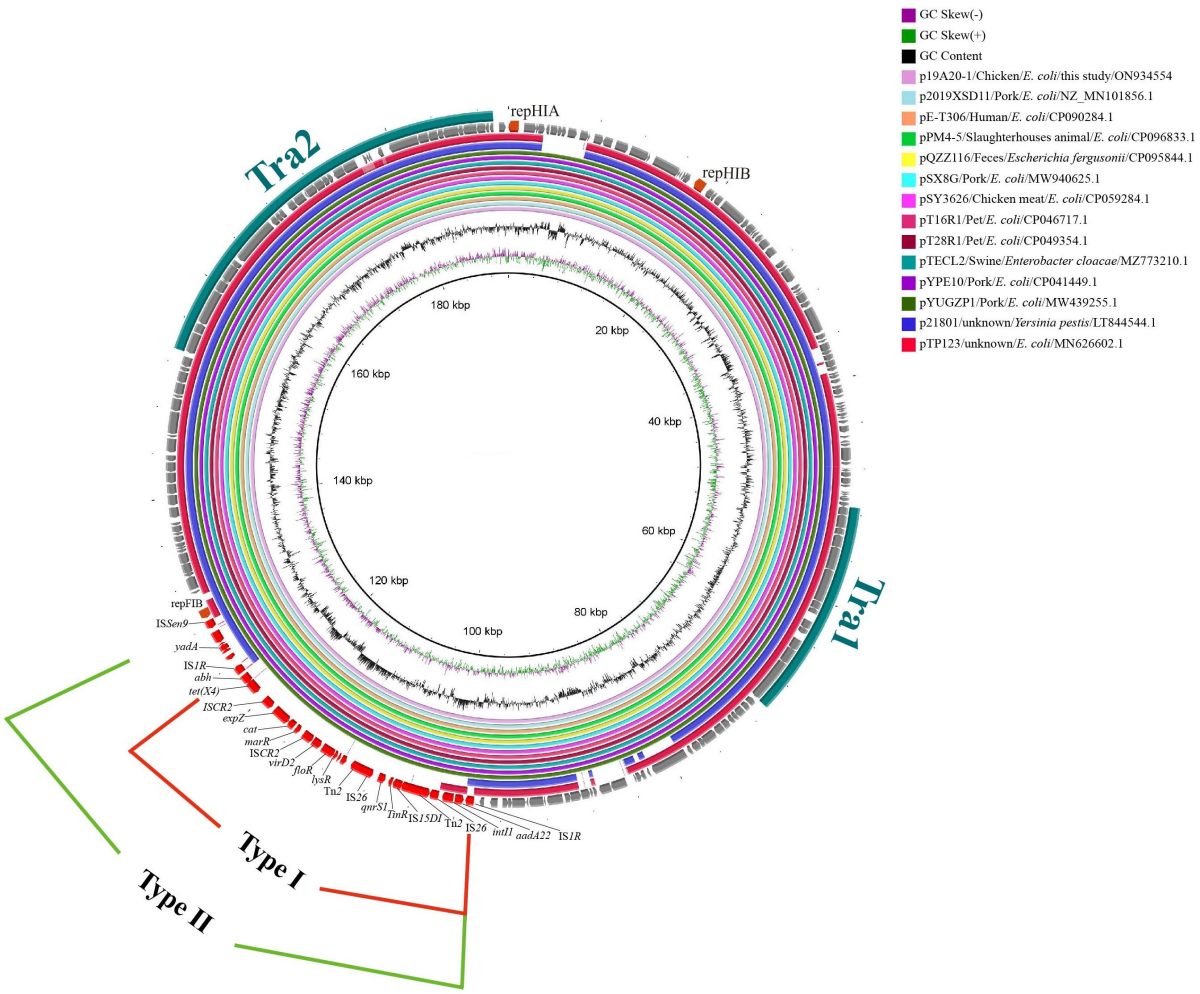
Circular alignment of *tet*(X4)-carrying IncHI1 plasmids. The tra region is colored turquoise. Gene positions and transcriptional directions in the outer circle were derived from p19A20, which was used as a reference. The red-solid box indicates the first case of variable region (25, 258 bp). The green-solid box indicates the second case of variable region (30, 056 bp).

**Figure 3:**
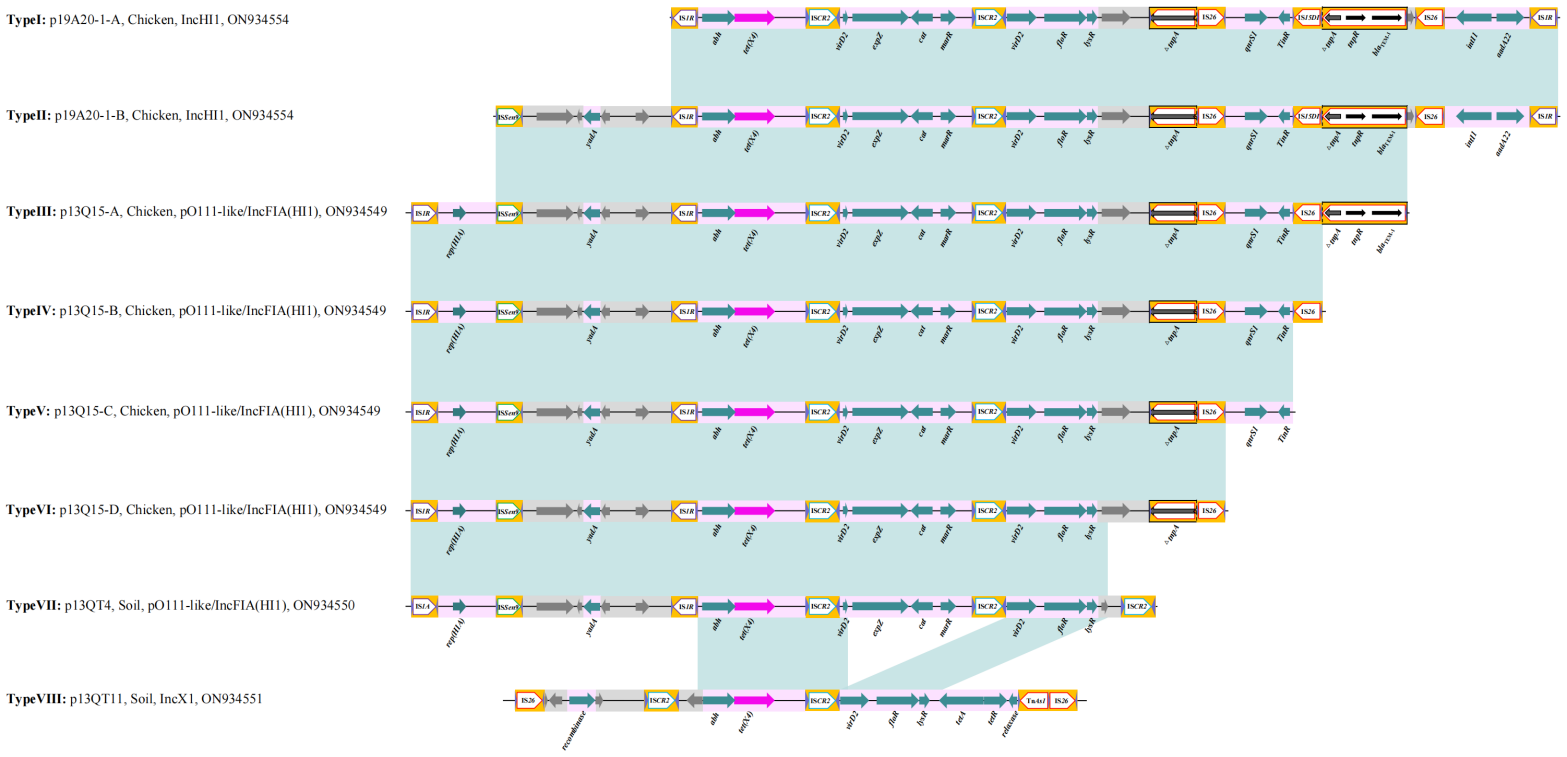
Summary of the different genetic environments of *tet*(X4) identified. Light blue shading indicates shared regions with a high degree of homology. Genes are represented by arrows. Bright purple represents *tet*(X4); turquoise represents resistant gene; white pentagon frame with yellow background represents Insert elements and transposons; gray represents hypothetical protein.

The three IncHI1 plasmids varied in size (from 190 to 244 kb) but there was more than 80% similarly in common regions. One noticeable difference is the BREX (Bacteriophage Exclusion, a novel phage resistance system that can block phage DNA replication) structure differentiating p19A20–1 (190.657 kb) and the other two (p13QT31–1, 212.2 kb; p22Q34–1, 244.406 kb) (Supplementary Figure S2). We found that p19A20–1 showed a high degree of similarity with other reference sequences (obtained from different hosts including breeding animals, pets, and humans) available in the NCBI database. It was clear that two distinct genetic structures that could mediate the transfer of *tet*(X4) were present in these related plasmids by comparing the complete sequence of p19A20–1 and 13 reference sequences (Figure 2). One 25,258 bp structure (IS*1R*-*abh*-*tet*(X4)-IS*CR2*-*orf2*-IS*CR2*-*orf3*-△Tn*2*-IS*26*-*orf4*-IS*15DI*-△Tn*2*-IS*26*-*orf5*-IS*1R*) containing multiple resistance genes (*tet*(X4), *floR*, *bla*_TEM-1_, *aadA22*, et al.) in one region flanked by IS*1R* was observed (Figure 3, TypeI). A larger variant of this structure was also observed with the ISS*en9*-hypothetical protein-*yadA*-hypothetical protein structure inserted upstream of the IS*1R*-*abh*-*tet*(X4)-IS*CR2* (Figure 3, TypeII).

**Figure 4:**
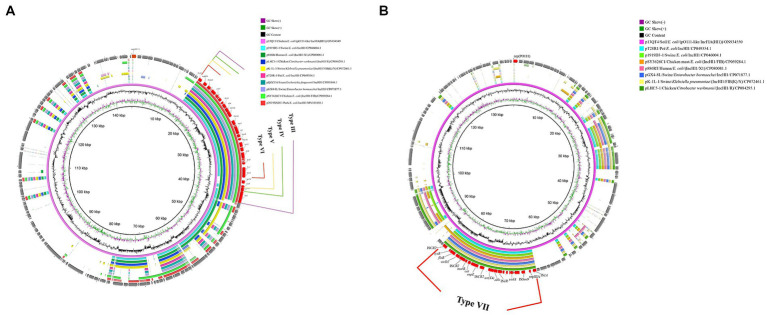
Circular alignment of *tet*(X4)-carrying pO111-like/IncFIA(HI1) plasmids. **(A)**: Gene positions and transcriptional directions in the outer circle were derived from p13Q15, which was used as a reference. Solid box of four colors indicates variable region (Type III 28,221 bp; Type IV 25,811 bp; Type V 25,004 bp; Type VI 23,044 bp). **(B)**: Gene positions and transcriptional directions in the outer circle were derived from p13QT4, which was used as a reference. A red box and the linear structure indicate the variable region (Type VII: 21,061 bp).

Further genetic environments (named here III to VII) of *tet*(X4) were also identified in pO111-like/IncFIA(HI1) plasmids. Four distinct genetic structures were identified by comparing the complete sequence of p13Q15 and other 9 reference plasmids. A core structure contained IS*1R*-rep (HIA)-IS*Sen9*-*hypothetical protein*-*yadA*-*hypothetical protein*-IS*1R*-*abh*-*tet*(X4)-IS*CR2*-△*virD2*-*expZ*-*cat*-*marR*-IS*CR2*-△*virD2*-*floR*-*lysR*-*hypothetical protein*-△*tnpA*-IS*26* (23,048 bp). Based on this structure, different variants (*qnrS1*-TinR, *qnrS1*-TinR-IS*26*, *qnrS1*-TinR-△*tnpA*-*tnpR*-*bla*_TEM-1_, respectively) were identified as inserted downstream of *tnpA*-IS*26* in the other three structures (Figure 4A, Figure 3, Type III, IV, V, VI). Only one *tet*(X4) organization was identified in p13QT4. The only major difference in this variant is the insertion of IS*CR2* downstream of the *lysr*-*hp* compared with p13Q15 and results in a truncated form of the Type VI structure with a loss of △*hypothetical protein*-△*tnpA*-IS*26* (Figure 4B; Figure 3, TypeVII).

**Figure 5:**
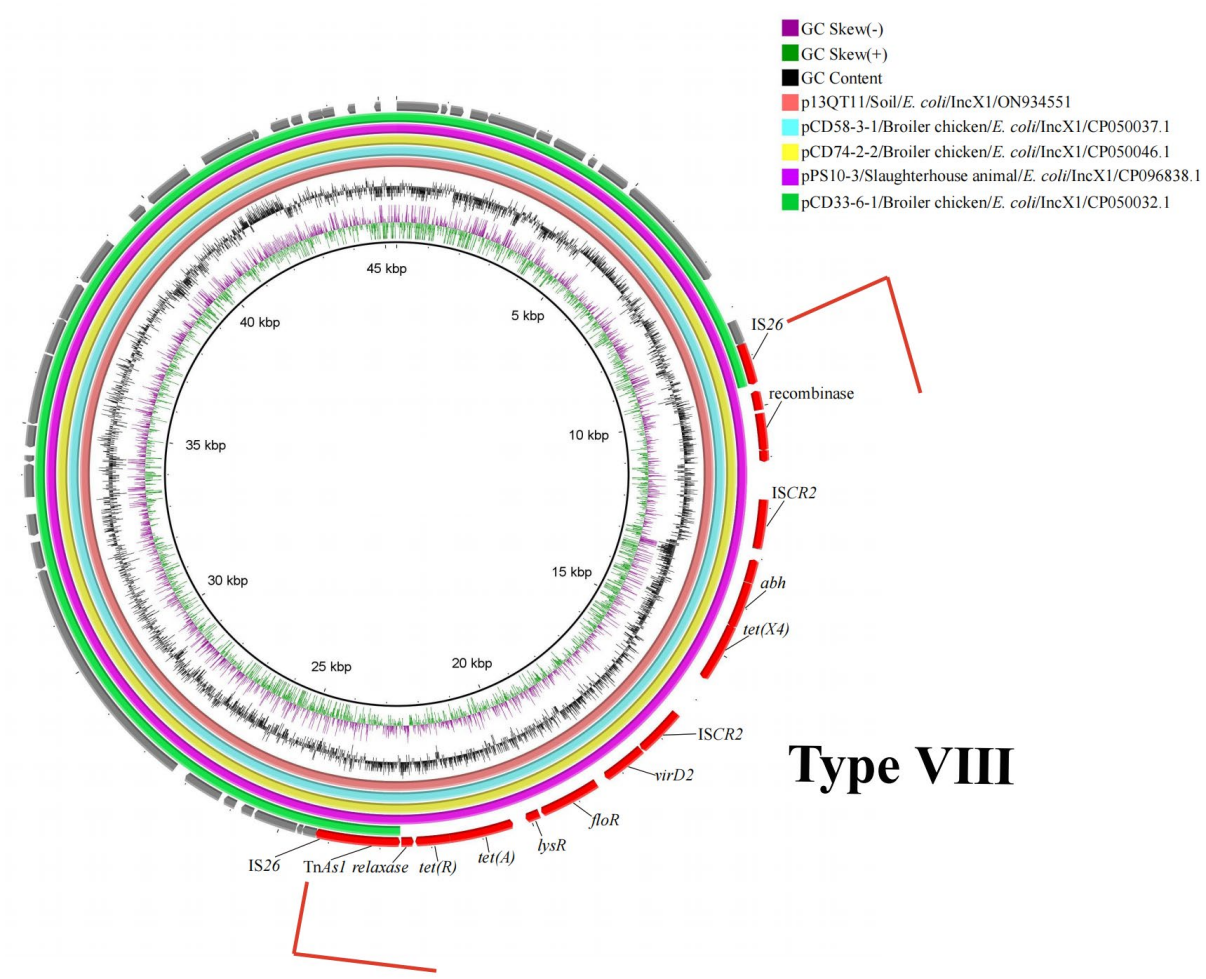
Circular alignment of *tet*(X4)-carrying IncX1 plasmids. Gene positions and transcriptional directions in the outer circle were derived from p13QT11, which was used as a reference. A red box and the linear structure indicate the genetic structure-containing *tet*(X4) (15,537 bp). The source of reference plasmids in the top right corner of circle. GeneBank accession no.: pCD58-3-1/NZ_CP050037.1; pCD74-2-2/NZ_CP050046.1; pPS10-3/NZ_CP096838.1; pCD33-6-1/NZ_CP050032.1.

The 8th genetic environment of *tet*(X4) confirmed in this study was found in IncX1 plasmids. Compared with reference plasmid pCD33-6-1, the remaining four plasmids (p13QT11, pCD58-3-1, pCD74-2-2, pPS10-3) carried the *tet*(X4) bearing genetic structure and the structure was an IS*26*-flanked (in the same orientation) transposition unit, IS*26*-*orf1*-IS*CR2*-*hypothetical protein*-*abh*-*tet*(X4)-IS*CR2*-*orf2*-Tn*As2*-IS*26* (15, 537 bp) (Figure 5). The upstream structure of *tet*(X4)-*abh* in p13QT11 showed significant inconsistencies compared to the other seven upstream structures. And the *virD2*-*expZ*-*cat*-*marR* structure, downstream of *tet*(X4)-IS*CR2* were found to have complete deletion in p13QT11 compared with genetic environments of IncHI1 and pO111-like/IncFIA(HI1) plasmids found in this study (Type VII).

## Discussion

Isolates of *E. coli* carrying *tet*(X4) have been reported in pigs, cows, ducks, sheep, migratory birds, and humans to date with the detection rate being less than 5% (Chen et al., 2020; Zhang et al., 2020a; Yu et al., 2021). The isolation rate of *tet*(X4)-positive *E. coli* in this study is much higher than that reported in previous studies. It is interesting that the positive rate of *tet*(X4)-harboring *E. coli* isolated from soil was also much higher when compared with fecal samples. Therefore, soil may be an important repository of *tet*(X4) resistant genes and the flow of strains between animals and the farm environment may provide a route for *tet*(X4) resistant gene transmission. Animal feces and the farm environment has been considered an important reservoir of antibiotic resistance genes. Animal feces can be used as fertilizer in agricultural production, which could aid transmission of *tet*(X4). The *tet*(X4) resistant gene would then be able to access a wide range of hosts, including strains presebt in agricultural soil and edible produce (He et al., 2023). Tetracycline agents of the earlier generations have been widely used in veterinary medicine worldwide and this has been suggested to be an important cause of the wide spread of tigecycline resistance in agricultural systems (Sun C. et al., 2019). The *tet*(X4)-positive *E. coli* in this study also carried other antibiotic resistance genes, consistent with the high rate of MDR in this study and this observation is consistent with several other studies (Sun J. et al., 2019b; He et al., 2021; Lu et al., 2021, 2022; Marathe et al., 2021; Sun et al., 2021; Feng et al., 2022).

A high genetic diversity of *E. coli* strains harboring *tet*(X4) in this study was detected by PFGE suggesting that the wide distribution of *tet*(X4) is mediated by horizontal genetic transfer rather than expansion of a single predominant clone. The transmission of *tet*(X4) in farming systems has become increasingly serious in recent years, including many food-producing animals (Sun C. et al., 2019; Lu et al., 2022; Wang et al., 2022). Previous studies have reported a diverse range of plasmids mediating transmission of *tet*(X4). We found that IncHI1 plasmids were by far the most common isolated from tigecycline resistant strains carrying *tet*(X4) in this study and have played the most important role in the transmission of *tet*(X4) in this setting. This is similar to recent evidence showing transferable *tet*(X4)-carrying IncHI1 plasmids were isolated from pork and duck farms (Bai et al., 2019; Yu et al., 2021). IncHI1 plasmids are potential vectors for the dissemination of genes among bacterial species in soil and aqueous environment (Phan and Wain, 2008). The *tet*(X4)-bearing IncHI1 plasmids were highly transferable and demonstrated stability in host strains in this study. The stability and the high transfer efficiency of IncHI1 plasmids observed could contribute to both high rates of *tet*(X4) transmission but also stable existence in the environment and gives further evidence for the potential of IncHI1 plasmids as a major vector mediating transmission of resistant genes in farms. We also discovered two additional families of plasmids, IncX1 and pO111-like/IncFIA(HI1) carrying *tet*(X4). To the best of our knowledge, pO111-like/IncFIA(HI1) is a novel family of plasmid carrying *tet*(X4). While we found the dominant vector mediating widespread of *tet*(X4) in this study to be IncHI1 plasmids we also believe that the gradual diversification of vector types might facilitate their interaction with a broad range of hosts (Villa et al., 2010). The IncX1 and pO111-like/IncFIA(HI1) plasmids did not possess autonomous transfer ability compared with IncHI1 plasmids which may help explain the dominance of IncHI1 plasmids seen in this study.

Insertion elements lead to the gradual formation of complicated genetic structures and promote the transfer of resistant genes. The IS*CR2* has been found next to resistance mechanisms in gram-negative organisms (Toleman et al., 2006). The special transposition model of IS*CR2* is crucial during the integration of *tet*(X4) into different types of plasmids and facilitates the chances of *tet*(X4) to spread among different bacterial species and expand its host range (Sun et al., 2020). The conserved region of *tet*(X4)-genetic environment has previously been largely reported as IS*CR2*-*tet*(X4)-*abh*, we expanded the region in this study and documented more complex *tet*(X4)-carrying structures containing multiple resistant genes and insertion elements. We found that IS*1R/A*, IS*Sen9*, IS*26* insertion elements also played a key role when extending the analysis for the genetic background of *tet*(X4). These insertion elements carry other resistant genes such as *floR*, *qnrS1* et al. and concomitant with *tet*(X4), which causes multi-resistant genes co-transfer. This can lead to multidrug-resistant bacterial infections occurring in more hosts. This greater complexity of insertion elements reflects the increased genetic diversification of *tet*(X4) evolution. It will also bring about more possibilities and opportunities for spread of *tet*(X4).

In conclusion, we found that *E. coli* isolated from different areas across one large chicken farm exhibit a high prevalence of tigecycline resistance. *tet*(X4)-production is a primary mechanism of resistance to tigecycline and IncHI1 plasmids are common vectors mediating transfer of *tet*(X4) in a representative large farm which is a matter of concern. The genetic structure of *tet*(X4) appears to be evolving and becoming more complicated. Our results suggest a mechanistic basis for the high detection rate of *tet*(X4) in recent years and it emphasizes the potential risk of transmission of *tet*(X4)-positive strains in farming systems.

There was a close relation between human health and food-producing animals with antibiotic resistance and it constitute a growing threat to sustainable agriculture. Increased surveillance of the multidrug-resistant bacteria in agriculture is urgently needed, such as control for antibiotics use as feed additives. In addition, we should also increase monitoring of plasmid mediated transfer of resistant genes in farms and improve antibiotic regimens in poultry farms as soon as possible to limit the spread of resistant bacteria.

## Data availability statement

The datasets presented in this study can be found in online repositories. The names of the repository/repositories and accession number(s) can be found at: NCBI- p13Q15 (ON934549), p13QT4, (ON934550), p13QT11 (ON934551), p13QT31-1 (ON934552), p19A20-1 (ON934554), p22Q34-1, (ON934556).

## Author contributions

YZ, JZ and H-XJ conceived and designed the experiments. YZ, JZ, PC, YL, M-TC, and X-LX performed the experiments. YZ and JZ analyzed the data. R-YS uploaded the sequencing data to the NCBI. YZ, MW, and H-XJ wrote and revised the manuscript. All authors approved the final draft and contributed to the article and approved the submitted version.

## Funding

This work was funded by the National Natural Science Foundation of China (Grant No. 31972734) and the Local Innovative and Research Teams Project of Guangdong Pearl River Talents Program (Grant No. 2019BT02N054).

## Conflict of interest

The authors declare that the research was conducted in the absence of any commercial or financial relationships that could be construed as a potential conflict of interest.

## Publisher’s note

All claims expressed in this article are solely those of the authors and do not necessarily represent those of their affiliated organizations, or those of the publisher, the editors and the reviewers. Any product that may be evaluated in this article, or claim that may be made by its manufacturer, is not guaranteed or endorsed by the publisher.

## Footnotes

^1^ http://rast.nmpdr.org

^2^ https://cge.food.dtu.dk/services/ResFinder/

^3^ https://www-is.biotoul.fr/index.php

^4^ https://cge.food.dtu.dk/services/PlasmidFinder/
