# Supplementary material for: IncHI1 plasmids are epidemic vectors that mediate transmission of tet(X4) in Escherichia coli isolated from China
Source: Front Microbiol. 2023 May 25;14:1153139. doi: 10.3389/fmicb.2023.1153139 (PMC10248516; doi:10.3389/fmicb.2023.1153139)
Supplement: Supplementary file 1 [file Data_Sheet_1.pdf]

Supplementary Material

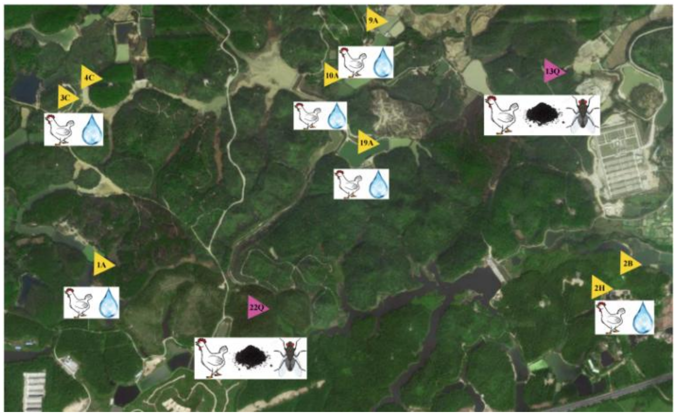

FIG S1 Map of sampling areas and sources in Chicken farm. The purple triangle line graph indicates the free-ranging region. The yellow triangle line graph indicates the intensive region. Patterns in white boxes represent the samples collected from chicken feces, soil and flies, respectively.

Table S1 The collection of samples and the detection of *tet*(X4)-carrying *Escherichia coli*.

Sample	Area of the farm	number of sampling (NS)[a]	Resistant to Tigecycline		*tet*(X4)-carrying *E. coli*		Selected for further research[d]
			Number (N_{TGC-R})[b]	N_{TGC-R}/NS (%)	Number ($N_{tet(X4)-C}$)[c]	$N_{tet(X4)-C}$/NS (%)	Number
Feces	1A	58	15	25.86	2	3.45	2
	9A	49	27	55.10	23	46.94	8
	10A	52	2	3.85	2	3.85	2
	19A	55	7	12.73	6	10.91	6
	2B	55	10	18.18	9	16.36	9
	3C	52	34	65.38	22	42.31	7
	4C	55	3	5.45	3	5.45	3
	2H	52	2	3.85	2	3.85	2
	13Q	50	18	36.00	16	32.00	4

	22Q	48	29	60.42	25	52.08	10
**Total**		**526**	**147**	**27.95**	**110**	**20.91**	**53**
Water	1A	2	0	0	0	0	0
	9A	5	0	0	0	0	0
	10A	5	0	0	0	0	0
	19A	5	0	0	0	0	0
	2B	5	0	0	0	0	0
	3C	5	0	0	0	0	0
	4C	5	0	0	0	0	0
	2H	5	0	0	0	0	0
	13Q	5	0	0	0	0	0
	22Q	5	0	0	0	0	0
**Total**		**47**	**0**	**0**	**0**	**0**	**0**
Soil	13QT	50	34	68.00	32	64.00	11
	22QT	31	23	74.19	23	74.19	8

**Total**		**81**	**57**	**70.37**	**55**	**67.90**	**19**
Fly	-	8	0	0	0	0	0
**Total (Feces, Water, Soil, Fly)**		**662**	**204**	**30.82**	**165**	**24.92**	**72**

a NS, sample number of collection from different regions; b N_{TGC-R}, number of tigecycline-resistant *E. coli*; c $N_{tet(X4)-C}$, number of *tet*(X4)-carrying *E. coli*; d Randomly selected strains for each region to further test.

Table S2 Basic information of 72 *tet*(X4)-carrying strains selected for the subsequent experiments.

Strain[a]	Sample	Area	*tet*(X4)-carrying plasmid	Plasmid size
1A39	Feces	1A	IncHI1	180
1A52	Feces		IncHI1	180
2B5	Feces	2B	IncHI1	190
2B13	Feces		IncHI1	200
2B16	Feces		IncHI1	190
2B17	Feces		IncHI1	200
2B21	Feces		IncHI1	190
2B26	Feces		IncHI1	190
2B32	Feces		IncHI1	190
2B34	Feces		IncHI1	190
2B47	Feces		IncHI1	180
2H40	Feces	2H	IncHI1	200
2H43	Feces		IncHI1	200
3C5	Feces	3C	IncHI1	190
3C14	Feces		IncHI1	190
3C21	Feces		IncHI1	190
3C23	Feces		IncHI1	200
3C28	Feces		IncHI1	200
3C38	Feces		IncHI1	190
3C46	Feces		IncHI1	190
4C35	Feces	4C	IncHI1	180
4C44	Feces		IncHI1	200
4C54	Feces		IncHI1	180
9A7	Feces	9A	IncHI1	190
9A9	Feces		IncHI1	230

9A10	Feces	9A	IncHI1	230
9A14	Feces		IncHI1	230
9A17	Feces		IncHI1	190
9A19	Feces		IncHI1	180
9A31	Feces		IncHI1	180
9A36	Feces		IncHI1	180
10A19	Feces	10A	IncHI1	190
10A41	Feces		IncHI1	200
13Q7-1	Feces	13Q	IncHI1	180
**13Q15**	Feces		pO111-like/IncFIA (HI1)	146
13Q22-1	Feces		IncHI1	180
13Q32-1	Feces		IncHI1	180
13QT2	Soil	13QT	IncHI1	180
**13QT4**	Soil		pO111-like/IncFIA (HI1)	137
**13QT11**	Soil		IncX1	45
13QT14	Soil		IncHI1	180
13QT17-	Soil		IncHI1	180
13QT22-	Soil		IncX1	45
13QT27	Soil		IncX1	45
**13QT31**	Soil		IncHI1	210
13QT38	Soil		IncHI1	190
13QT37-	Soil		IncHI1	210
13QT45-	Soil		IncHI1	210
19A2	Feces	19A	IncHI1	200
19A6	Feces		IncHI1	180
19A8	Feces		IncHI1	200
19A11	Feces		IncHI1	200
**19A20**	Feces		IncHI1	190

19A39	Feces		IncHI1	220
22Q3	Feces	22Q	IncHI1	200
22Q4	Feces		IncHI1	180
22Q7	Feces		IncHI1	190
22Q18	Feces		IncHI1	200
22Q19	Feces		IncHI1	200
22Q28	Feces		IncHI1	180
**<u>22Q34</u>**	Feces		IncHI1	244
22Q41	Feces		IncHI1	180
22Q46	Feces		IncHI1	200
22Q47	Feces		IncHI1	180
22QT1	Soil	22QT	IncHI1	180
22QT5	Soil		IncHI1	180
22QT6	Soil		IncHI1	200
22QT7	Soil		IncHI1	200
22QT8	Soil		IncHI1	180
22QT10	Soil		IncHI1	200
22QT16	Soil		IncHI1	180
22QT17	Soil		IncHI1	180

a: Underlined and bold fonts strains indicate were picked for illumina and nanopore sequencing.

Table S3 sequence accession numbers.

Strains	Plasmids[a]	accession number
13Q15	**p13Q15**	ON934549
13QT4	**P13QT4**	ON934550
13QT11	**P13QT11**	ON934551
13QT31	**p13QT31-1**	ON934552
	p13QT31-2	ON934553
19A20	**p19A20-1**	ON934554
	p19A20-2	ON934555
22Q34	**p22Q34-1**	ON934556
	p22Q34-1	ON934557

a: Underlined and bold fonts plasmids indicate that plasmids carry *tet*(X4) resistance gene.

Table S4 The conjugation frequency of IncHI1 plasmids isolated from the chicken farm.

Strain	Region	Source	Plasmid size(≈)	conjugation frequency
13Q32-1	13Q	Feces	180	5.49×10^{-3}
13QT38		Soil	190	6.72×10^{-2}
13QT14			180	1.71×10^{-1}
13QT45-1			210	6.70×10^{-2}
13QT17-1			104	1.0×10^{-7}
13QT31			210	3.67×10^{-2}
22Q41	22Q	Feces	180	5.12×10^{-4}
22Q7			190	1.09×10^{-3}
22Q34			244	2.65×10^{-4}
22QT1		Soil	180	9.2×10^{-2}
22QT6			200	3.4×10^{-1}
9A7	9A	Feces	190	7.1×10^{-2}
9A14			230	3.51×10^{-2}
9A10			230	1.34×10^{-2}
9A36			180	3.9×10^{-2}

19A20	19A	Feces	190	3.94×10^{-3}
19A39			220	4.95×10^{-2}
2B47	2B	Feces	180	3.65×10^{-3}
2B32			190	1.07×10^{-1}
3C21	3C	Feces	190	1.25×10^{-1}
3C23			200	4.63×10^{-2}
3C46			190	4.68×10^{-2}
4C54	4C	Feces	180	1.93×10^{-3}
4C44			200	3.42×10^{-3}
1A52	1A	Feces	180	1.56×10^{-4}
10A19	10A	Feces	190	1.0×10^{-7}
2H40	2H	Feces	200	2.28×10^{-4}

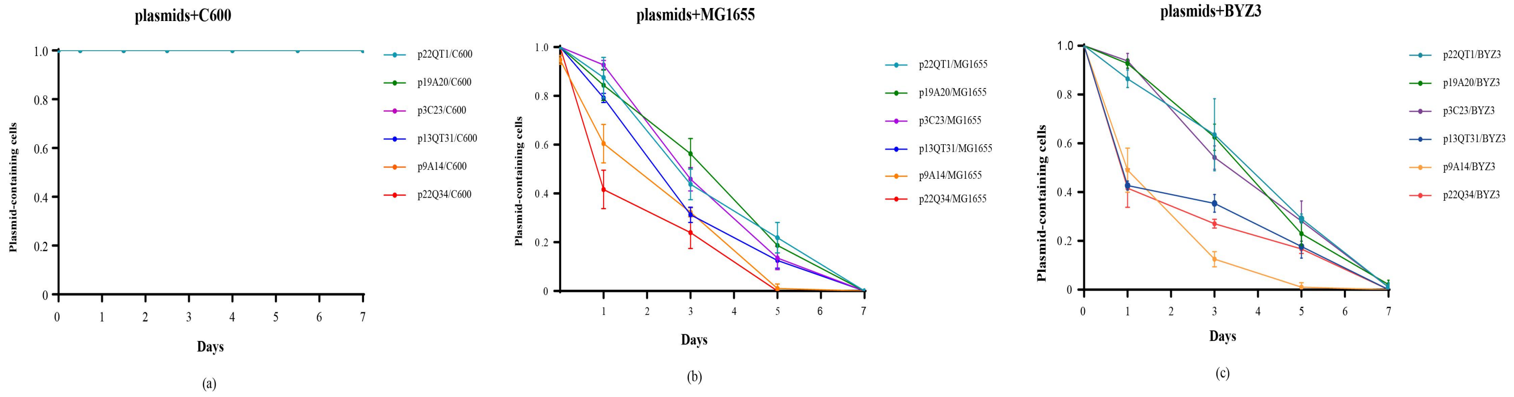

FIG S2 Stability determination of different IncHI1 plasmids in *E. coli* strain. Plasmids used in this study were named "p" as a prefix with the name of isolates. The six plasmids utilized were p22QT1 (≈180 kb), p19A20 (≈190 kb), p3C23 (≈200 kb), p13QT31 (≈210 kb), p9A14 (≈230 kb) and p22Q34 (≈244 kb). (a): the recipient strain was C600; (b): the recipient strain was MG1655; (c): the recipient strain was BYZ3.

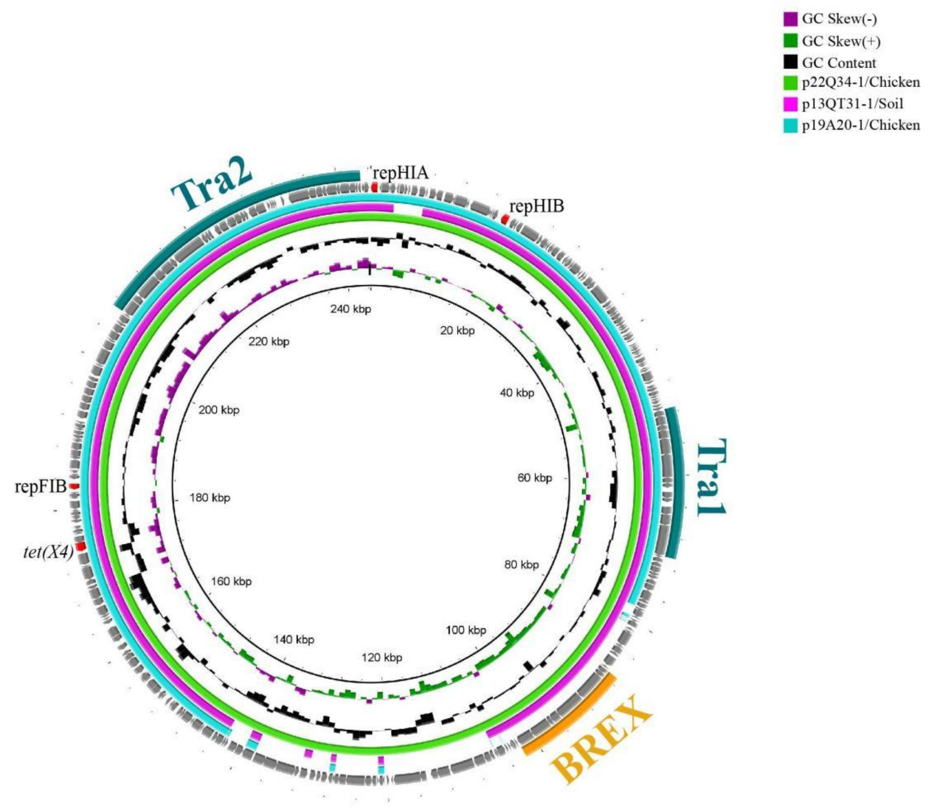

FIG S3 Circular alignment of *tet*(X4)-carring IncHI1 plasmids (p22Q34-1, p13QT31-1 and p19A20-1). The *tra* region is colored navy and BREX region is colored ginger. Gene positions and transcriptional directions in the outer circle were derived from p22Q34-1, which was used as a reference.
